# Low-Input, High-Resolution 5′ Terminal Filovirus RNA Sequencing with ViBE-Seq

**DOI:** 10.3390/v16071064

**Published:** 2024-07-01

**Authors:** Stephen J. Ross, Adam J. Hume, Judith Olejnik, Jacquelyn Turcinovic, Anna N. Honko, Lindsay G. A. McKay, John H. Connor, Anthony Griffiths, Elke Mühlberger, Daniel Cifuentes

**Affiliations:** 1 Department of Virology, Immunology & Microbiology, Chobanian and Avedisian School of Medicine, Boston University, Boston, MA 02215, USA; sjross@bu.edu (S.J.R.); hume@bu.edu (A.J.H.); jolejnik@bu.edu (J.O.); jturcino@bu.edu (J.T.); honko@mac.com (A.N.H.); lmckay@bu.edu (L.G.A.M.); jhconnor@bu.edu (J.H.C.); ahgriff@bu.edu (A.G.); 2 National Emerging Infectious Diseases Laboratories, Boston University, Boston, MA 02215, USA; 3 Department of Biochemistry & Cell Biology, Chobanian and Avedisian School of Medicine, Boston University, Boston, MA 02215, USA

**Keywords:** 5′ end RNA sequencing, ViBE-Seq, viral bona fide end sequencing, emerging viruses, Ebola virus, Marburg virus, filovirus, terminal deoxynucleotidyl transferase

## Abstract

Although next-generation sequencing (NGS) has been instrumental in determining the genomic sequences of emerging RNA viruses, de novo sequence determination often lacks sufficient coverage of the 5′ and 3′ ends of the viral genomes. Since the genome ends of RNA viruses contain the transcription and genome replication promoters that are essential for viral propagation, a lack of terminal sequence information hinders the efforts to study the replication and transcription mechanisms of emerging and re-emerging viruses. To circumvent this, we have developed a novel method termed ViBE-Seq (Viral Bona Fide End Sequencing) for the high-resolution sequencing of filoviral genome ends using a simple yet robust protocol with high fidelity. This technique allows for sequence determination of the 5′ end of viral RNA genomes and mRNAs with as little as 50 ng of total RNA. Using the Ebola virus and Marburg virus as prototypes for highly pathogenic, re-emerging viruses, we show that ViBE-Seq is a reliable technique for rapid and accurate 5′ end sequencing of filovirus RNA sourced from virions, infected cells, and tissue obtained from infected animals. We also show that ViBE-Seq can be used to determine whether distinct reverse transcriptases have terminal deoxynucleotidyl transferase activity. Overall, ViBE-Seq will facilitate the access to complete sequences of emerging viruses.

## 1. Introduction

Emerging and re-emerging zoonotic viruses are an increasing threat to public health. This is exemplified by the Ebola virus (EBOV) outbreak in West Africa in 2014, the emergence of the Nipah virus in Bangladesh and India, and the recent SARS-CoV-2 pandemic [1,2,3,4]. The preemptive surveillance of potential animal reservoirs of emerging viruses has led to the discovery of novel viruses with potential for future zoonotic spillover [5,6,7]. Many of these novel viruses are closely related to known human pathogens. These include the newly discovered filoviruses, Lloviu virus, Mengla virus, and Dehong virus as well as the novel henipaviruses, including Ghana virus, Mojiang virus, and Langya henipavirus [8,9,10,11,12]. With few exceptions, these viruses have been detected solely by high-throughput sequencing methods while no infectious viral particles have been isolated from either animal hosts or infected individuals. Unfortunately, fragmentary sequencing data and a lack of virus isolates hampers the possibility to study their mechanisms of replication, assess their pathogenicity, and develop antiviral countermeasures.

Emerging filoviruses of concern have proven challenging to study due to the difficulty of isolating infectious viral particles from field samples, the lack of appropriate cell lines for virus propagation, and the requirement for work in high-containment laboratories. Useful tools to study these viruses include reverse genetics systems to generate recombinant viral clones as well as viral replicon systems that mimic crucial steps of the viral replication cycle and can be used at biosafety level 2 (BSL-2) [13,14,15,16,17,18]. The development of any of these systems relies on the accurate characterization of the complete viral genomic sequence including the genome ends, which contain sequences crucial to filovirus replication and transcription, polymerase binding, and genome packaging. Therefore, determining the precise sequence of the viral genome ends is critical for the study of these viruses [19,20].

High-throughput genomic sequencing methods are generally successful in de novo identification of a substantial portion of novel filoviral genome sequences, but often fail to provide sufficient coverage of the genomic 5′ and 3′ ends [21,22,23,24,25]. Due to the use of random priming in large-scale de novo cloning methods, the chances to convert genomic RNA to cDNA diminish with increasing proximity to the genome ends. A more recent approach, nanopore direct RNA sequencing, has been instrumental in determining mRNA sequences and transcriptional start sites, but the requirement of a poly(A) tail in the substrates to be sequenced using standard nanopore protocols prevents the sequencing of genomes lacking polyadenylation. Other methods such as 5′ Rapid Amplification of cDNA Ends (RACE) require the ligation of an oligonucleotide adaptor at the 5′ end of the viral RNA, but ligation efficiency decreases in complex RNA mixtures [26]. Overall, there is an unmet need for orthogonal, streamlined methods that can be used to determine the sequence of filoviral RNA ends with high fidelity, little processing, and limited amounts of RNA from field samples.

Here we report the development of ViBE-Seq (Viral Bona Fide End Sequencing), a novel method aimed at precise sequence identification of the genomic, antigenomic, and mRNA 5′ ends of filoviruses to complement existing methods. To validate this method, we used the highly pathogenic EBOV and Marburg virus (MARV) as our test viruses. We have successfully sequenced the 5′ ends of both EBOV and MARV genomic RNA using as little as 50 ng of total RNA isolated from virions, infected cells, or animal tissue. Finally, we determined the 5′ end of the EBOV nucleoprotein (NP) mRNA to precisely map and analyze transcription start sites.

ViBE-Seq capitalizes on the circularization of the cDNA to avoid pre-processing and potential degradation of the template RNA, effectively preserving the identity of the 5′ end of the RNA. Finally, the seamless integration of ViBE-Seq with high-throughput sequencing protocols allows for precise sequence identification.

## 2. Materials and Methods

### 2.1. Biosafety Statement

All work with EBOV and MARV was performed in the biosafety level 4 (BSL-4) facility of Boston University’s National Emerging Infectious Diseases Laboratories (NEIDL) following approved standard operating procedures (SOPs) in compliance with local and national regulations pertaining to handling BSL-4 pathogens and Select Agents [27].

### 2.2. Cell Culture

Vero E6 cells (African green monkey kidney cells; ATCC CRL-1586) were maintained in Dulbecco’s modified Eagle medium (DMEM; Gibco/Thermo Fisher Scientific, Waltham, MA, USA) supplemented with 10% fetal bovine serum (FBS; R&D Systems, Minneapolis, MN, USA), 2 mM L-glutamine (Thermo Fisher Scientific, Waltham, MA, USA), and either 100 U/mL penicillin-streptomycin (Thermo Fisher Scientific, Waltham, MA, USA) or 100 μg/mL Primocin (Invivogen, San Diego, CA, USA). Cells were grown at 37 °C and 5% CO_2_.

### 2.3. Virus Propagation

EBOV Mayinga and MARV Musoke isolates were kindly provided by Heinz Feldmann, NIH NIAID Rocky Mountain Laboratories, Hamilton, MT, USA. Virus stocks were propagated in Vero E6 cells in cell culture medium (DMEM supplemented with 2% FBS and 100 μg/mL Primocin). After a post-infection incubation time of 5 to 10 days, or when decent cytopathic effects were visible, cell supernatants were clarified by low-speed centrifugation. For purified stocks, viral particles were purified by ultracentrifugation through a 20% sucrose cushion as previously described [28]. Virus titers were determined in Vero E6 cells either by 50% tissue culture infectious dose (TCID_50_) assay and calculated using the Spearman–Kärber algorithm or by plaque assay. For the nonhuman primate study, an EBOV Kikwit stock generated by Public Health England (Ebola virus H.sapiens-tc/COD/1995/Kikwit-9510621, lot HCM2018/30) was used for infection. This isolate from the original human sample was passaged three times in Vero E6 cells (P3 stock).

### 2.4. Animal Tissue

Only banked tissue samples were used in this study. Rhesus macaques were infected intramuscularly with 100 plaque-forming units (PFU) of EBOV Kikwit as part of an unrelated study. Samples were collected at necropsy from moribund animals that were euthanized between 6 and 7 days post-exposure. Sections from the right lateral liver were retained at <−60 °C until processed.

### 2.5. Viral Sequences

The following NCBI reference sequences were used for sequence analysis: Zaire ebolavirus isolate Ebola virus/H.sapiens-tc/COD/1976/Yambuku-Mayinga (GenBank accession number NC_002549), Zaire ebolavirus isolate Ebola virus/H. sapiens-tc/COD/1995/Kikwit-9510621 (GenBank accession number KU182905.1), Marburg marburgvirus isolate Marburg virus/H.sapiens-tc/KEN/1980/Mt. Elgon-Musoke (GenBank accession number NC_001608).

### 2.6. Analysis of Reverse Transcriptase Terminal Deoxynucleotide Transferase Activity

A total of 100 ng of an EBOV minigenome plasmid containing the 5′ of the EBOV genome [29] was used as a template for PCR using Q5 PCR reaction (New England Biolabs, Ipswich, MA, USA). A primer containing a T7 RNA polymerase (T7) promoter was used to create a 400-nucleotide EBOV 5′ end DNA template for in vitro transcription. Once amplified, the amplicon was resolved by agarose gel electrophoresis and gel-purified using the Monarch DNA Gel Extraction Kit (New England Biolabs, Ipswich, MA, USA). Then, 500 ng of purified product was used for in vitro transcription using the MEGAscript T7 Transcription Kit (Thermo Fisher Scientific, Waltham, MA, USA). RNA was purified from the reaction by acid phenol:chloroform extraction followed by isopropanol precipitation as performed in Section 2.10. The resulting RNA was used as a template for ViBE-Seq and subsequent terminal nucleotide analysis.

### 2.7. RNA Isolation

#### 2.7.1. Virion RNA

Then, 2.2 × 10^8^ TCID_50_ units of sucrose cushion-purified EBOV particles in phosphate buffered saline (PBS; Thermo Fisher Scientific, Waltham, MA, USA) were inactivated by adding 3 volumes of TRIzol LS (Thermo Fisher Scientific, Waltham, MA, USA) following approved SOPs. An amount of 0.25 mL of MARV stock in cell culture medium containing 2.7 × 10^6^ TCID_50_ units of infectious virus were inactivated by adding 0.75 mL of TRIzol LS. Total RNA was purified according to the manufacturer’s instructions and quantified using a Nanodrop spectrophotometer (Thermo Fisher Scientific, Waltham, MA, USA).

#### 2.7.2. Total Cellular RNA from Infected Cells

Vero E6 cells were seeded in a 6-well plate at 5 × 10^5^ cells per well the day prior to infection. Cells were infected with EBOV isolate Mayinga at a multiplicity of infection of 5, 24 h post infection, and 1 mL of TRIzol (Thermo Fisher Scientific, Waltham, MA, USA) was added to each well. Cells resuspended in TRIzol were collected in 2 mL O-ring capped tubes and inactivated following approved SOPs. Total RNA was purified according to the manufacturer’s instructions and quantified using a Nanodrop spectrophotometer.

#### 2.7.3. Total Cellular RNA from Infected NHP Liver Tissue

Frozen tissue samples were thawed, and ≤100 mg of tissue was added to a tube containing a 5 mm stainless steel bead and 1 mL of TRIzol reagent. Samples were homogenized using a TissueLyser II (Qiagen, Germantown, MD, USA) by two rounds of 2 min at 30 Hz. Total RNA was extracted according to the manufacturer’s instructions and quantified using a Nanodrop spectrophotometer.

### 2.8. Reverse Transcription

The Sensiscript Reverse Transcription Kit (Qiagen, Germantown, MD, USA) was used to reverse transcribe 50 ng of virion RNA, total cellular RNA isolated from infected cells, or total cellular RNA isolated from infected liver tissue according to the manufacturer’s instructions. The Omniscript Reverse Transcription Kit (Qiagen, Germantown, MD, USA) was used to reverse transcribe 200 ng of total RNA from infected cells according to the manufacturer’s instructions. In either case, reactions were incubated at 37 °C for 2 h. Reverse transcription adaptor sequences are provided in Supplementary Table S1.

### 2.9. cDNA Marker Synthesis

We synthesized a single-stranded (ss) DNA fragment of the expected size of the generated cDNA products to be used as a molecular weight marker during size fractionation: 100 ng of an eGFP expression vector was used as a template for a Q5 PCR reaction. Reactions were run as follows: 98 °C (1 min), 98 °C (10 s), 65 °C (30 s), 72 °C (12 s), repeat 40×, 72 °C (2 min), 4 °C (hold). PCR products were analyzed by gel electrophoresis on a 2% agarose gel. Gel portions were excised corresponding to either 200 or 230 base pairs to match the expected target viral sequence length. Products were purified using the Monarch DNA Gel Extraction Kit according to the manufacturer’s instructions. The purified PCR products were then used as a template for Lambda exonuclease reaction according to the manufacturer’s instructions (1 μL enzyme) to degrade 5′-phosphorylated DNA strands, leaving the unphosphorylated strand intact as ssDNA. Single-stranded cDNA products were separated on a 10% denaturing polyacrylamide gel and purified as described in Section 2.10.

### 2.10. cDNA Purification

Reverse transcription products and a cDNA marker were separated on a 10% denaturing polyacrylamide TBE-urea gel. To prepare the gel, 5 mL of the UreaGel Buffer, 20 mL of the UreaGel Concentrate, and 25 mL of the UreaGel Diluent (National Diagnostics, Atlanta, GA, USA) were mixed in a 50 mL conical Falcon tube. Then, 500 μL of 10% ammonium persulfate (Thermo Fisher Scientific, Waltham, MA, USA) and 50 μL of TEMED (Thermo Fisher Scientific, Waltham, MA, USA) were added to the mixture before casting the gel. After running the samples for 2 h at 12 Watts constant, the gel was stained with SYBR Gold (Thermo Fisher Scientific, Waltham, MA, USA) for 10 min by shaking at room temperature. Stained gels were visualized under blue light and the bands corresponding to the expected cDNA product size were excised. Gel fragments were pulverized by extrusion through holes punched in a 0.5 mL microcentrifuge tube with a 21.5 gauge needle and centrifugation at 21,000× *g* for 5 min. The pulverized polyacrylamide was collected in 1.5 mL Eppendorf microcentrifuge tubes and resuspended into a slurry with 500 μL of 0.4 M NaCl solution. Tubes were shaken overnight at 4 °C and 2000 rpm in an Eppendorf Thermomixer to facilitate cDNA elution. Polyacrylamide was then removed from the slurry via centrifugation in a barrier microcentrifuge tube (COSTAR; Thermo Fisher Scientific, Waltham, MA, USA) at 21,000× *g* for 5 min. Then, 1 μL of Glycoblue Co-precipitant (Thermo Fisher Scientific, Waltham, MA, USA) and 1 volume of 100% isopropanol was added to the resulting solution. cDNA was allowed to precipitate overnight at −20 °C. The cDNA was pelleted via centrifugation at 21,000× *g* for 1 h at 4 °C. Isopropanol was aspirated from the tube and the cDNA pellet was washed with 1 mL of 80% ethanol. The cDNA was centrifuged again at 21,000× *g* for 15 min at 4 °C and the ethanol was aspirated. The cDNA pellet was allowed to air-dry for 5 min after which the cDNA was resuspended in 20 μL of nuclease-free water. cDNA was quantified using a Nanodrop spectrophotometer.

### 2.11. cDNA Circularization

Between 3 and 5 pmol of purified cDNA was circularized using CircLigase ssDNA ligase I (Biosearch Technologies, Beverly, MA, USA). Reactions were conducted according to the manufacturer’s instructions. Briefly, reactions were incubated for 2 h at 60 °C and 10 min at 80 °C. The final cDNA concentration was quantified via Nanodrop spectrophotometer.

### 2.12. Target Sequence Amplification

Endpoint PCR was used to amplify the viral sequences from the circularized cDNA. Primer sequences are provided in Supplementary Table S1.

To amplify the viral 5′ end sequences, 60 ng of circularized cDNA was used as a template for a two-cycle Q5 PCR reaction using the following conditions: 98 °C (1 min); 98 °C (10 s), 43 °C (20 s), 72 °C (30 s) for 5–6 cycles; 98 °C (10 s), 55–67 °C (20 s), 72 °C (30 s), 40 cycles, 72 °C (2 min); 4 °C (hold). PCR products were separated on a 2% agarose gel. DNA bands were excised and purified using the Monarch DNA Gel Extraction Kit (New England Biolabs, Ipswich, MA, USA) according to the manufacturer’s instructions. Purified products were combined as necessary to obtain 5–10 ng DNA/μL in 35 μL of nuclease-free water.

### 2.13. Sequencing

Technical replicates of purified PCR products were pooled and submitted to Mass General Hospital’s Center for Computation and Integrative Biology DNA Core in Cambridge, MA, for cloning and sequencing using Illumina instruments. Sequencing FASTQ files provided by the core were obtained with ~100,000 reads.

### 2.14. Sequencing Result Analysis

FASTQ files generated by Mass General Hospital’s DNA core were checked for quality using FASTQC [30]. Adaptors were trimmed using cutadapt [31]. FASTQ files were aligned to viral reference sequences using bowtie2. Resulting .bam and .tsv files were generated with samtools view and samtools depth, respectively [32]. Sequencing duplicates were removed with picard leaving 80–100% of reads for analysis [33]. Intact 5′ ends and terminal nucleotides were quantified with the BBMAP suite from reads containing the EBOV 5′ end sequence 5′ ACACACAAAAAAGAAGAA 3′ or the MARV 5′ end sequence 5′ ACACACAAAAAAGAUGAA 3′ [34]. Reverse complement sequences were generated with FASTX-toolkit. FASTQ files were converted to FASTA format with Seqtk v1.4. Alignments were generated with MAFFT [35]. Alignment results were graphed using R and RStudio (2023). Internal single nucleotide variants were quantified with LoFreq v2 [36].

## 3. Results

### 3.1. ViBE-Seq Utilizes cDNA Circularization to Amplify Target Sequences

ViBE-Seq relies on the circularization of cDNA to avoid unnecessary ligations of adaptors, similar to the cloning procedures used in ribosomal profiling and Cross-linking and Immunoprecipitation (CLIP) strategies [37,38]. ViBE-Seq also avoids pre-processing of the viral RNA template including poly(A) tailing, de-phosphorylation, or mRNA de-capping to lessen the extent of RNA manipulation and maximize the amount of template available for reverse transcription.

**Figure 1:**
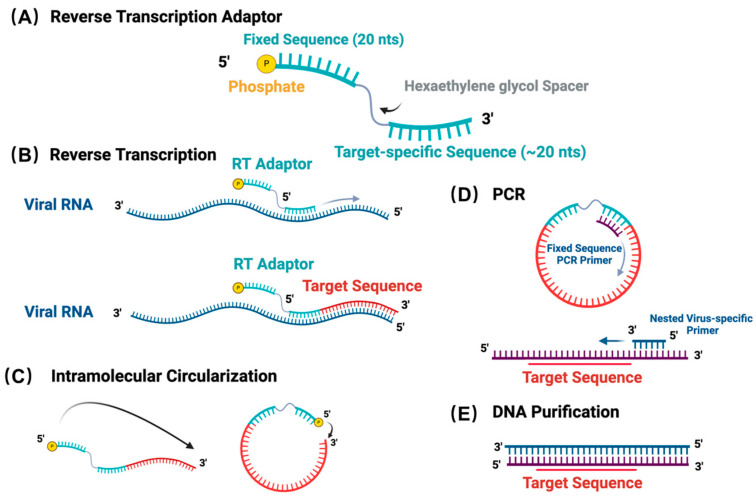
Workflow of the ViBE-Seq protocol. (**A**) Schematic of the general reverse transcription adaptor structure. (**B**) Reverse transcription of viral RNA target sequence using phosphorylated reverse transcription adaptor. (**C**) Intramolecular circularization of target sequence and phosphorylated RT adaptor following cDNA purification. (**D**) Polymerase chain reaction with a nested set of primers. (**E**) DNA purification with a product containing the target 5′ end of viral RNA.

The reverse transcription (RT) adaptor utilized in ViBE-Seq has a modular design to allow target-sequence amplification, cDNA circularization, and primer binding during PCR (Figure 1A). To streamline efforts, we have developed ViBE-Seq to facilitate reverse transcription of the 5′ end of the viral RNA directly from isolated RNA without prior RNA processing. The target-specific adaptor binds within the last portion of the known sequence of the viral 5′ end (Figure 1B). To be able to sequence the entire fragment with a set of paired-end Illumina sequencing reads of 150 nucleotides each, this sequence was placed approximately 200–230 nucleotides upstream of the target 5′ end. However, we anticipate that amplicons of larger sizes will work as well depending on the cloning and sequencing specifications. Once synthesized, the RT adaptor allows for intramolecular circularization of the captured cDNA via a terminal 5′ phosphate. This 5′ phosphate facilitates the intramolecular ligation of the 3′ end of the newly synthesized cDNA strand to the 5′ end of the adaptor, creating a single stranded, circular cDNA product containing the complete viral 5′ end (Figure 1C). This step was designed to avoid ligating external adaptors to target RNAs in complex mixtures and instead perform this ligation step on cDNA. Moreover, the ligation that occurs in ViBE-Seq is intramolecular rather than intermolecular, which aims to increase ligation efficiency that may suffer from non-specific ligations in existing methods [26]. The fixed sequence and the target-specific sequence within the adaptor provide templates for primer binding during PCR amplification (Figure 1D). Importantly, the fixed sequence is used solely as a primer binding site and can be replaced by, in theory, any sequence. The hexaethylene glycol spacer placed between the fixed sequence and the target-specific sequence prevents the production of any rolling circle amplicons (Figure 1A). Additionally, this spacer also contains a restriction enzyme site if the cDNA product needs to be linearized for any downstream processes (Supplementary Table S1). 

Following reverse transcription, the target cDNA product is resolved by electrophoresis on a 10% denaturing polyacrylamide gel to purify the cDNA of the expected size. The purified cDNA is then used as a template for circularization by CircLigase I. After this step, PCR is used to amplify the target sequence using a semi-nested virus-specific primer. This primer should overlap with the 3′ terminus of the RT adaptor and a known portion of the target sequence (Figure 1D). This semi-nested primer is essential to increasing target sequence specificity and amplifying the correct cDNA template. PCR products are analyzed on a 2% agarose gel and bands corresponding to the expected sizes are excised, purified, and sent for sequencing (Figure 1E). It is worth mentioning that in attempting to uncover unknown viral termini, phylogenetic analysis and sequence alignments can offer an inference for the length of the missing sequence [13].

The total working time of this protocol is two to three days, in addition to the time it takes to sequence the final product. We show the amenability of ViBE-Seq to different types of viral RNA sources, including those from tissue samples from infected animals.

### 3.2. An Amount of 50 ng of Purified Virion RNA Is Sufficient for 5′ End Sequence Determination

**Figure 2:**
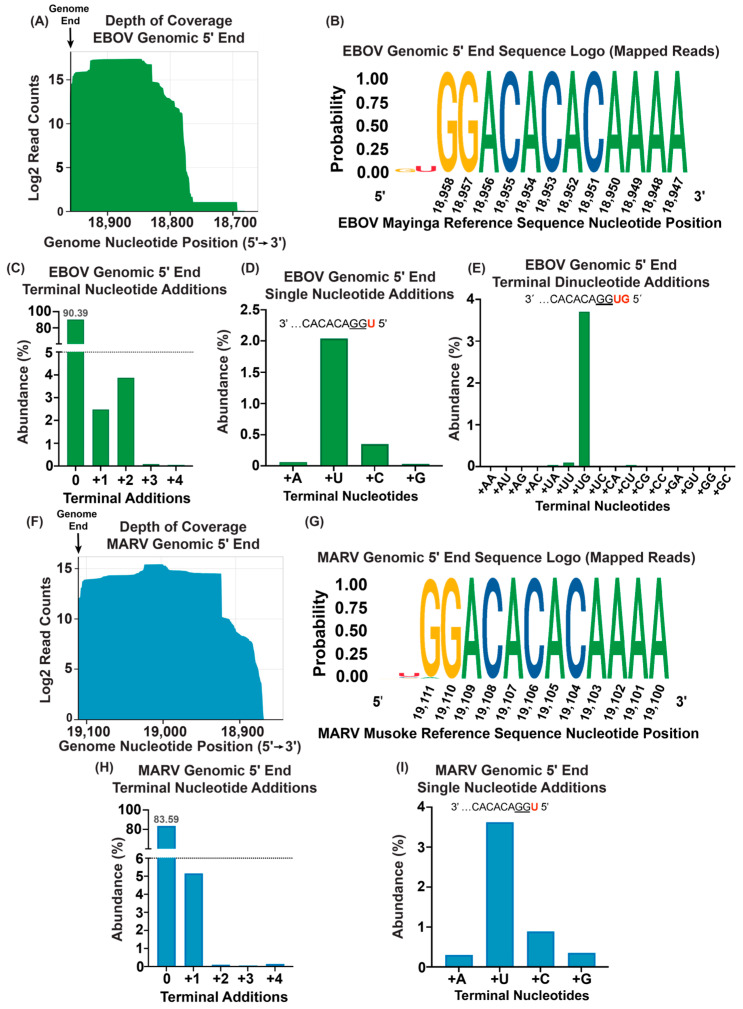
Mapping the genomic 5′ end of EBOV and MARV using virion RNA. (**A**) Depth of sequencing coverage of the 5′ end of the EBOV genome. The vertical black line depicts the published 5′ end of the viral genomes. Results are graphed in a Log2 scale. (**B**) Sequence logo of anchored reads mapped to the EBOV genomic 5′ end. Genomic sequence shown in 5′ to 3′ orientation. (**C**) Percent abundances of terminal nucleotide additions observed in reads mapped to the EBOV genomic 5′ end. (**D**) Percent abundances of 5′ single-nucleotide additions from (**C**). The most abundant single-nucleotide addition shown in red. (**E**) Percent abundances of EBOV genomic 5′ terminal dinucleotide additions from (**C**). The most abundant dinucleotide addition shown in red. (**F**) Depth of sequencing coverage of the 5′ end of the MARV genome. (**G**) Sequence logo of reads mapped to the MARV genomic 5′ end. Genomic sequence shown in 5′ to 3′ orientation. (**H**) Percent abundances of terminal nucleotide additions in reads mapped to the MARV genomic 5′ end. (**I**) Percent abundances of 5′ single-nucleotide additions from (**H**). The most abundant single-nucleotide addition is shown in red.

Purified virion-derived RNA represents a relatively homogenous RNA population. We therefore used EBOV and MARV genomic RNA purified from virions to first test the suitability of ViBE-Seq for determining the 5′ ends of viral genomic RNA. Following the ViBE-Seq protocol with only 50 ng of input RNA, we were able to capture nucleotide-resolution sequencing data for the complete genomic 5′ end of EBOV. With these data, we were able to visualize significant depth of genomic coverage to the very last nucleotide of the 5′ end (Figure 2A). Consensus analysis of sequences mapped to the EBOV reference sequence revealed little variation of the annotated 5′ end with low abundance of 5′ terminal additions (Figure 2B). Specifically, 90.39% of total EBOV-specific sequences contained an intact 5′ end with no additional nucleotides. In total, 2.48% of the sequenced EBOV 5′ ends contained one additional nucleotide, with a U residue being the most abundant nucleotide addition, and 3.88% contained two additional nucleotides: UG, UU, UA, and CU (Figure 2C–E). Less than 0.1% percent of the RNA population contained three or four additional nucleotides and were not included in the downstream analysis (Figure 2C). Other EBOV-specific sequences contained truncations that spanned the final 5′ nucleotides. 

We also obtained nucleotide-resolution sequencing data for the complete genomic 5′ end of MARV. Again, we were able to visualize a significant depth of coverage through the last nucleotide of the MARV genomic 5′ end (Figure 2F). Consensus analysis of sequences mapped to the MARV reference sequence revealed little variation to the annotated 5′ end with 1.3% of sequences containing an A residue at nucleotide 19,111 instead of a G (Figure 2G). In total, 83.59% of the sequences mapped to the MARV reference sequence contained an intact 5′ end with no terminal nucleotide additions. However, 5.16% of the sequences contained one additional nucleotide. Only <1% of the reads contained two–four additions which were not considered in downstream analyses due to their low abundance (Figure 2H). Of the low abundance sequences that contained one additional nucleotide at the 5′ end, the majority (3.62%) contained an additional U residue with minor populations showing an additional C (0.89%), G (0.35%), or A (0.30%) residue (Figure 2I). 

### 3.3. Viral Genome and mRNA 5′ Termini Captured from RNA Isolated from Infected Cells

**Figure 3:**
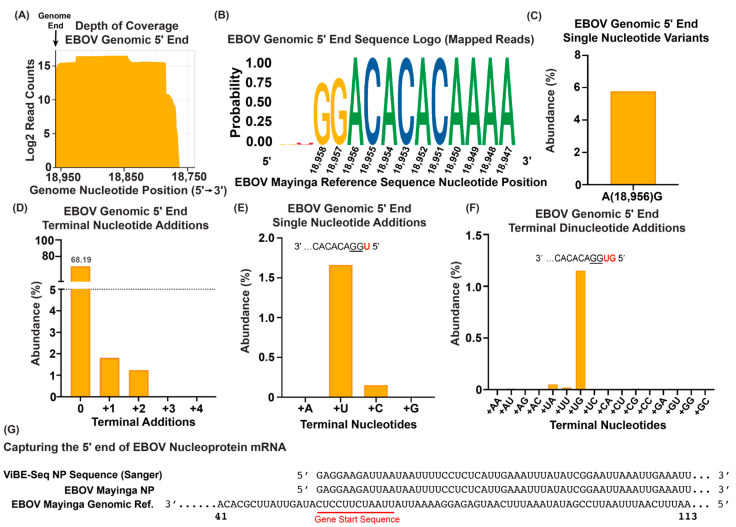
Capturing EBOV genome and mRNA 5′ termini using total RNA isolated from infected cells. (**A**) Depth of sequencing coverage of the 5′ end of the EBOV genomic from infected Vero E6 cells. The vertical black line depicts the published 5′ end of the viral genomes. Results are graphed in a Log2 scale. (**B**) Sequence logo of anchored reads mapped to the EBOV genomic 5′ end. Genomic sequence shown in 5′ to 3′ orientation. (**C**) Percent abundance of sequence variation within the genomic 5′ end region observed in mapped reads (min. depth of 10 and frequency > 0.5). (**D**) Percent abundances of 5′ terminal nucleotide additions observed in reads mapped to the EBOV genomic 5′ end. (**E**) Percent abundances of 5′ single-nucleotide additions from (**D**). The most abundant single-nucleotide addition shown in red. (**F**) Percent abundances of EBOV genomic 5′ terminal dinucleotide additions from (**D**). The most abundant dinucleotide addition shown in red. (**G**) Sanger sequencing results of the ViBE-Seq protocol capturing the 5′ end of EBOV NP mRNA. The red line depicts the NP gene start signal. “Reference” abbreviated to “Ref.”

We next explored whether ViBE-Seq was suitable for determining the 5′ ends of viral RNAs isolated from EBOV-infected cells. This approach aimed at validating ViBE-Seq’s sensitivity in complex mixtures of both cellular and viral RNAs. Total RNA isolated from EBOV-infected Vero E6 cells 24 h post-infection was used as a template for ViBE-Seq using Sensiscript (50 ng) or Omniscript (200 ng) reverse transcriptase. We were successful in obtaining nucleotide-resolution sequencing data for the complete EBOV genomic 5′ end despite the high abundance of non-template RNA species (Figure 3A). A consensus analysis of these sequences revealed little variation in the annotated 5′ end with a low abundance of 5′ terminal additions (Figure 3B). Single-nucleotide variants analyzed with LoFreq revealed that 5.8% of mapped sequences contained an internal A to G mutation at nucleotide 18,956 that was not present in the data derived from virion RNA (Figure 3C) [36]. Compared with the ViBE-Seq results obtained with virion RNA, we observed a higher frequency of truncations prior to the 5′ terminal GG residues (Figure 3B). Only 68.19% of the EBOV sequences isolated from infected cells contained an intact trailer with no terminal nucleotide additions. Less than 2% of the intact EBOV genome sequences contained one or two terminal nucleotide additions. We did not observe sequence reads that contained three or more terminal nucleotide additions (Figure 3D). Similar to what was observed for virion RNA (Figure 2D,E), the most abundant single-nucleotide addition was a U residue (1.66%) and the most frequent dinucleotide addition was UG, followed by UA and UU (Figure 3E,F). To gauge ViBE-Seq’s utility to positive-strand RNA, we next aimed to capture the 5′ end of the EBOV nucleoprotein (NP; first gene) mRNA. Using total RNA isolated from EBOV-infected cells, we were able to capture the 5′ end of the EBOV NP mRNA. In this case, the sequence of the ViBE-Seq product was determined by Sanger sequencing (Figure 3G). This result validates that ViBE-Seq can be used to determine the 5′ ends of both positive-sense and negative-sense viral RNAs. Also, Sanger sequencing may be useful as a read-out if significant sequence variation is not expected in the final product.

### 3.4. ViBE-Seq Captures EBOV 5′ Genome Ends from Infected Animal Tissue

**Figure 4:**
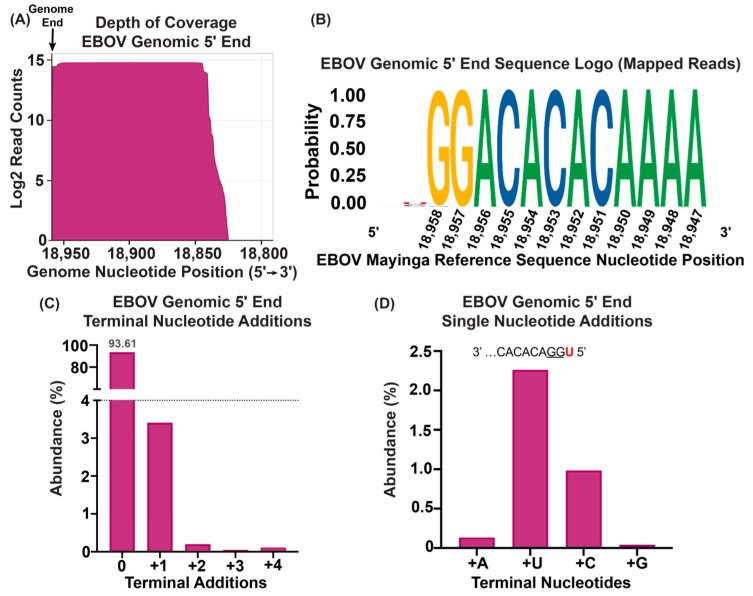
Capturing EBOV genome 5′ termini using total RNA isolated from liver tissue obtained from infected animals. (**A**) Depth of sequencing coverage of the 5′ ends of the EBOV genome using 50 ng of total RNA isolated from liver tissue. Results graphed in a Log2 scale. The black line depicts the 5′ end of the viral genome. (**B**) Sequence logo of anchored reads mapped to the EBOV genomic 5′ end. Genomic sequence shown in 5′ to 3′ orientation. (**C**) Percent abundances of terminal nucleotide additions observed in reads mapped to the EBOV genomic 5′ end. (**D**) Percent abundances of 5′ single-nucleotide additions from (**C**).

While cell culture provides an accessible platform to model viral infection, infected animal tissue is often the primary source of viral RNA obtained from field work. Therefore, we explored if animal tissue could be used as source material for ViBE-Seq. An amount of 50 ng of total RNA isolated from EBOV-infected non-human primate liver tissue was used in ViBE-Seq using Sensiscript reverse transcriptase. Although ex vivo tissue represents the most complex sample that we utilized as input, we again observed nucleotide-resolution sequencing data for the complete EBOV genomic 5′ end with considerable coverage (Figure 4A). Surprisingly, we observed the highest frequency of intact EBOV genomes compared with virion and cellular RNA. In total, 93.61% of the sequences mapped to the EBOV reference sequence contained an intact 5′ end with no terminal nucleotide additions (90.39% for virions; 68.19 for cell culture samples). A consensus analysis of the sequences mapped to the EBOV reference sequence revealed little variation in the annotated 5′ end with a low abundance of 5′ terminal additions. Notably, 0.5% of sequences contained a C residue at nucleotide 18,958 instead of a G which was not observed for virion and cell culture RNA (Figure 4B). Only 3.41% of the mapped sequences showed a terminal single-nucleotide addition with a U residue being the most abundant addition, and 0.2% or less contained two–four terminal nucleotide additions (Figure 4C,D). These results show that ViBE-Seq represents a high-fidelity approach to sequence viral genomic 5′ ends from infected animal biopsies.

### 3.5. ViBE-Seq Captures Terminal Deoxynucleotidyl Transferase Activity of RT Polymerases

Throughout our analyses, we observed the presence of low-abundance non-templated nucleotides at the 5′ end of the analyzed viral genomes. Our results suggest a bias in the abundance of these untemplated nucleotides, with U residues being most prominent, followed by C, G, and A. Previous work by us suggested that the EBOV genomic 5′ end does not contain untemplated nucleotides [39]. Therefore, we sought to reveal the origin of these untemplated nucleotides.

**Figure 5:**
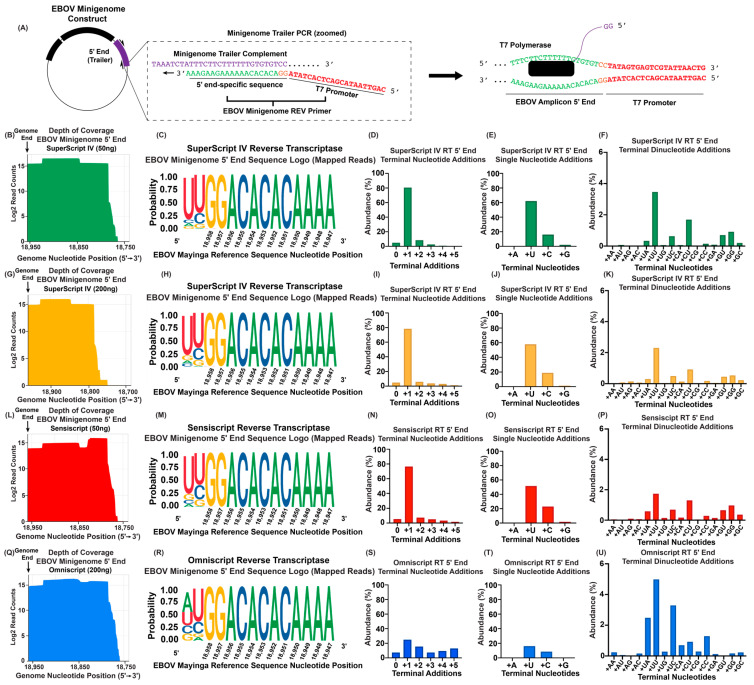
Using ViBE-Seq to determine terminal transferase activity of various reverse transcriptases. (**A**) Schematic of PCR primer containing T7 promoter and in vitro RNA synthesis of the EBOV minigenome 5′ end. Orange GG in REV primer denotes the overlap between T7 promoter and genome terminus. (**B**) Depth of sequencing coverage of the 5′ end of the EBOV minigenome trailer cDNA using SuperScript IV reverse transcriptase with 50 ng of input RNA. The vertical black line depicts the published 5′ end of the viral genome. Results are graphed in a Log2 scale. (**C**) Sequence logo of anchored reads mapped to the EBOV genomic 5′ end. Genomic sequence shown in 5′ to 3′ orientation. (**D**) Percent abundances of terminal nucleotide additions observed in reads mapped to the EBOV genomic 5′ end. (**E**) Percent abundances of 5′ single-nucleotide additions from (**C**). (**F**) Percent abundances of 5′ terminal dinucleotide additions from (**C**). (**G**) Depth of sequencing coverage of the 5′ end of the EBOV minigenome trailer cDNA using SuperScript IV reverse transcriptase with 200 ng of input RNA. (**H**) Sequence logo of anchored reads mapped to the EBOV genomic 5′ end. Genomic sequence shown in 5′ to 3′ orientation. (**I**) Percent abundances of terminal nucleotide additions in reads mapped to the EBOV genomic 5′ end. (**J**) Percent abundances of 5′ single-nucleotide additions from (**I**). (**K**) Percent abundances of EBOV genomic 5′ terminal dinucleotide additions from (**I**). (**L**) Depth of sequencing coverage of the 5′ end of the EBOV minigenome trailer cDNA using Sensiscript RT with 50 ng of input RNA. The vertical black line depicts the published 5′ end of the viral genome. Results are graphed in a Log2 scale. (**M**) Sequence logo of anchored reads mapped to the EBOV genomic 5′ end. Genomic sequence shown in 5′ to 3′ orientation. (**N**) Percent abundances of terminal nucleotide additions observed in reads mapped to the EBOV genomic 5′ end. (**O**) Percent abundances of 5′ single-nucleotide additions from (**N**). (**P**) Percent abundances of EBOV genomic 5′ terminal dinucleotide additions from (**N**). (**Q**) Depth of sequencing coverage of the 5′ end of the EBOV minigenome trailer cDNA using Omniscript RT with 200 ng of input RNA. (**R**) Sequence logo of anchored reads mapped to the EBOV genomic 5′ end. Genomic sequence shown in 5′ to 3′ orientation. (**S**) Percent abundances of terminal nucleotide additions in reads mapped to the EBOV genomic 5′ end. (**T**) Percent abundances of 5′ single-nucleotide additions from (**S**). (**U**) Percent abundances of EBOV genomic 5′ terminal dinucleotide additions from (**S**).

Reverse transcriptases have a reported propensity to add 3′ non-templated nucleotides during cDNA synthesis due to their terminal deoxynucleotidyl transferase (TdT) activities. During analysis, these nucleotides are indistinguishable from bona fide 5′ terminal nucleotides in the input RNA. To investigate background levels of terminal nucleotide addition, we utilized ViBE-Seq with our previous conditions and commonly used reverse transcriptases using an RNA with a defined 5′ end. A 400-nucleotide DNA fragment spanning the EBOV genomic 5′ end was amplified by PCR using an EBOV minigenome plasmid as a template. One of the used primers contained a T7 promoter. The resulting amplicon encoding the EBOV 5′ end sequence fused to a T7 promoter was confirmed by Sanger sequencing (Figure 5A) (Supplementary Data S1; Supplementary Table S1). This amplicon was in vitro transcribed, and the resulting RNA was used as a template for ViBE-Seq, providing a well-defined template for 5′ end analysis. Note that the sequences shown in Figure 5 have been converted to their reverse complement to reflect the correct orientation of the EBOV genomic 5′ end RNA sequence as used in all previous figures. This means that the reported terminal nucleotide additions were synthesized as their complement at the cDNA level. For example, Moloney murine leukemia virus (MMLV)-based reverse transcriptases have a propensity to add untemplated nucleotides to cDNA with a bias of A > G > C > T [40]. In this report, this would be shown as U > C > G > A.

Since it was previously reported that MMLV-based reverse transcriptases have TdT activity, we used the MMLV-based SuperScript IV reverse transcriptase as a control for this experiment with the expectation that SuperScript IV would show TdT activity. In parallel, we analyzed potential TdT activities of the non-MMLV reverse transcriptases, Sensiscript and Omniscript (both Qiagen, Germantown, MD, USA). 

Using 50 ng of in vitro transcribed RNA for cDNA synthesis with SuperScript IV reverse transcriptase, we successfully obtained cDNA that, upon sequencing, mapped to the EBOV genomic 5′ end including the 5′ terminal GG residues (Figure 5B,C). Intriguingly, only a small percentage (4.89%) of the intact sequences showed no terminal nucleotide additions, while 80.47% of this population contained one additional terminal nucleotide and 8.44% contained two additional terminal nucleotides (Figure 5D). Regarding the single-nucleotide additions, 62.03% of the sequences showed an additional U residue (A residue at cDNA level), 16.13% an additional C residue, 2% a G residue, and 0.3% an A residue, confirming the untemplated nucleotide bias previously reported for MMLV-based reverse transcriptases [40] (Figure 5E). Importantly, these nucleotides were not present within the template amplicon (Supplementary Table S1; Supplementary Data S1). We also captured a low percentage of various dinucleotide additions with UU dinucleotides being most frequently observed (3.46%) (Figure 5F). To control for higher amounts of input RNA, the ViBE-Seq protocol was repeated with SuperScript IV with an input amount of 200 ng of RNA. The obtained results were similar to those observed with 50 ng input RNA (Figure 5G−K). Together, this shows that the ViBE-Seq protocol was suitable to recapitulate the previous reports of TdT activity and terminal deoxynucleotide bias of a MMLV-based reverse transcriptase. 

Next, we analyzed the TdT activity of non-MMLV reverse transcriptases, including Sensiscript and Omniscript. Using 50 ng of input RNA, Sensiscript reverse transcriptase successfully synthesized the complete EBOV 5′ genomic end with considerable coverage (Figure 5L,M). Terminal nucleotide analysis of intact genome end sequences revealed that only 5.26% of this population had no untemplated nucleotide additions, while the majority of these sequences (76.37%) contained a single-nucleotide addition (Figure 5N). Two to five nucleotide additions were observed for a smaller percentage of reads (1.73–7.26%) (Figure 5N). A total of 51.51% of the single-nucleotide additions were identified as a U residue, 22.99% as a C, 1.6% as a G, and 0.26% as an A (Figure 5O). Sequences containing two additional nucleotides were not abundant, with the most enriched containing a terminal UU dinucleotide at 1.74% (Figure 5P). These data suggest that Sensiscript reverse transcriptase has considerable TdT activity with a similar nucleotide bias to that found for the SuperScript IV enzyme (Figure 5B–K).

Omniscript reverse transcriptase requires higher quantities of input RNA for reverse transcription than Sensiscript. We used 200 ng of input in vitro transcribed RNA to obtain nucleotide-resolution sequencing data of the cDNA spanning the complete EBOV 5′ genome end (Figure 5Q,R). Although the percentage of reads containing one extra nucleotide (24.97%) was diminished compared with the data obtained with the Superscript and –reverse transcriptases, it was still considerably higher than the number of reads without any nucleotide addition (7.3%) (Figure 5S). Omniscript reverse transcriptase seemed to be more efficient in adding two–five nucleotides in abundances ranging from 7.34% to 15.48% (Figure 5S). Again, a majority of the +1 sequences contained one additional U residue (16.03%), followed by C (8.49%), while G and A residues made up 0.29% and 0.15%, respectively (Figure 5T). Similar to the data obtained using Sensiscript, a terminal UU dinucleotide was the most abundant two-nucleotide addition (4.98%) (Figure 5U). Together, these data show that ViBE-Seq can be used to determine reverse transcriptase TdT activity and that the reverse transcriptases used in this study have considerable TdT activity.

## 4. Discussion

With ViBE-Seq, we have developed a novel high-throughput method that allows for the direct sequencing of cDNA ends derived from filovirus RNAs. With single-nucleotide resolution, this protocol allows for the rapid detection of genomic 5′ end variation within a viral population or between distinct infected samples. We demonstrated the robustness of ViBE-Seq for capturing intact filoviral genomic 5′ termini using viral RNA derived from virions, infected cells, and tissue from infected animals. While the protocol is amenable to higher amounts of RNA, it has proven highly sensitive and successful in capturing complete sequences with as little as 50 ng of input RNA in both simple virion-derived and complex cellular- and tissue-derived RNA mixtures. We believe this makes ViBE-Seq the protocol of choice for determining the actual genome ends of recombinant and emerging filoviruses, especially when only minute amounts of RNA are available for processing. ViBE-Seq can be used as a high-throughput, orthogonal approach to complement existing methods such as 5′ RACE (Supplementary Table S1). We previously used this protocol to sequence the genomic 5′ ends of recombinant Lloviu virus, an emerging member of the filovirus family [17]. Although we have only applied ViBE-Seq to filoviruses, we anticipate that this protocol could also be used for determining the genome ends of other negative- and positive-sense RNA viruses.

Using both Sensiscript and Omniscript, we were able to capture the complete 5′ end sequences of viral RNA species derived from two distinct filoviruses and various RNA sources. Using virion RNA as the input, we determined that the majority of intact EBOV genomic 5′ ends contained no additional nucleotide at the 5′ end with the majority of the genomes captured terminating in GG. Our results provide little evidence of the terminal U residue annotated in the EBOV reference sequence. Our previous work demonstrated that EBOV 5′ terminal U or UG residues are rare and do not reflect most of the virus population [39]. However, it has to be noted that many EBOV genome sequences posted in GenBank contain an additional 5′ U residue [41]. The remaining single-nucleotide and dinucleotide additions identified in this study fell below a frequency of 2%. While we report low-abundance variants as the output of our method, we believe these hold little significance due to the potential contribution of reverse transcriptase activity or artifacts of the sequencing method. We obtained similar results for the 5′ ends of MARV genomes purified from virions. The vast majority of sequences terminated in GG, with less than 5% of sequences showing a single-nucleotide addition. Since our results are consistent with previously published work, we conclude that the majority of genomes from the purified virions we used here terminate with GG and do not contain significant amounts of RNA species with additional nucleotides.

Viral RNA extracted from infected cells is commonly used to analyze infection and virus evolution. Here, we determined both EBOV genomic and NP mRNA 5′ ends from cellular RNA isolated from EBOV-infected cells, confirming that ViBE-Seq can be employed for the analysis of both negative and positive sense RNA obtained from various sources. Compared with virion RNA, we observed a significant reduction in the number of full-length sequences covering the complete EBOV genomic 5′ end (Figure 3B,D). This could be due to degradation during the cloning process or reduced replication fidelity in infected cells. As was also observed for virion RNA, there was little evidence of nucleotide additions to the 5′ terminal GG residues. Interestingly, only the EBOV genomic RNA obtained from infected cells showed some sequence variability for nucleotide 19,856 (Figure 3C; 5.8% A to G transition). It is not clear if this is due to procedure-specific variability or cell culture adaptation.

Often, the only material available to identify emerging viruses are animal biopsies collected during fieldwork. We were able to capture the EBOV genome 5′ end sequence using 50 ng of RNA isolated from the liver tissue of infected animals, effectively expanding the source of samples that can provide input RNA for ViBE-Seq. We also saw little evidence of 5′ terminal nucleotide additions. Interestingly, RNA isolated from infected animal tissue contained the highest quantity of intact EBOV genomic 5′ ends compared with virion RNA and RNA isolated from infected cells. This might be attributed to procedure-specific differences during RNA isolation or the increased stability of viral RNA in animal tissue compared with cultured cells [42].

It was previously reported that MMLV-based reverse transcriptases, such as SuperScript IV, have TdT activity with a bias for adding A residues to the 3′ ends of the cDNA with a frequency ranging between 25 and 90% [40]. Similarly, it is known that MMLV-based reverse transcriptases add non-templated nucleotides to up to 80% of clones generated in 5′ RACE experiments [40]. Our study confirms this finding and shows the same trend for Sensiscript reverse transcriptase. The origin of Sensiscript has not been disclosed. According to the Sensiscript manual (Qiagen, Germantown, MD, USA), it is a recombinant enzyme different from MMLV and avian myeloblastosis virus reverse transcriptases. Omniscript, which was isolated from equine infectious anemia virus [43], appears to add a single untemplated nucleotide at a much lower frequency. RNA species with longer non-templated nucleotide additions (>3 nucleotides) were observed at a higher frequency with Omniscript compared with the other reverse transcriptases. To our knowledge, this is the first characterization of the TdT activity of both Sensiscript and Omniscript. 

Our findings using in vitro synthesized RNA (Figure 5) emphasize that one must be aware of the potential contribution of reverse transcriptase TdT activity when analyzing RNA 5′ ends. It may be necessary to create a threshold for tolerated non-templated nucleotides based on these results. 

It is also important to note that we observed considerable differences in the abundance of untemplated terminal nucleotides in the in vitro transcribed RNA controls and the viral RNA samples. The reason for these differences is not immediately clear. A possible explanation for these unexpected results includes the high number of short RNA molecules in the in vitro reaction, as weighed by mass to match the input mass of the virion and total RNA mixtures. It is conceivable that the overabundance of short template RNAs promotes TdT activity. However, this conclusion requires more investigation. Of note, we and others have previously used Sensiscript reverse transcriptase to analyze the 5′ ends of viral RNAs by primer extension and did not observe considerable TdT activity [39,44]. This emphasizes the need to characterize TdT-like activity of reverse transcriptases more carefully and in various conditions for unique applications. 

A limitation of ViBE-Seq is the reliance on an accurate length estimate for the target 5′ end. If the length of the missing 5′ end is unknown, we recommend comparing the target viral sequence to reference sequences of related viruses to design a target-specific primer within the desired amplicon range. Due to ViBE-Seq’s reliance on cDNA purification from a gel, it is important to know the approximate length of the expected cDNA for successful gel excision. This could make it difficult to capture 5′ terminal sequences of unknown lengths. In developing the ViBE-Seq protocol, we attempted column cDNA purification as well as excising incremental portions of the entire gel with little success and a high background during amplification. Therefore, this method is optimized for sequence identification with an educated estimate of the target sequence’s length. To determine the 5′ ends of uncharacterized viral RNA genomes, we highly recommend a phylogenetic analysis in comparison with closely related viruses. This analysis should provide enough information for an educated approximation of 5′ end lengths. This strategy has been successfully employed to infer the 3′ end of the Lloviu virus genome [13]. If this is insufficient, cDNA excision from the gel at increasing sizes will cover all potential lengths. 

In summary, we have developed a protocol to determine the precise 5′ ends of filoviral RNAs isolated from virions, infected cells, or animal tissue using as little as 50 ng of total RNA. Our low input RNA requirement could make ViBE-Seq the method of choice to determine the 5′ genome ends of known or newly emerging filoviruses obtained from field samples [45,46]. Of note, many genome sequences of newly discovered viruses lack their terminal nucleotides, and even for well-known viruses, the published genome ends might be erroneous. For example, the results of this study in conjunction with previous work present a robust case to correct the current reference EBOV genome sequence to indicate the correct 5′ genomic end sequence, GG. Overall, with this novel methodology, we hope to streamline the process of viral sequence determination and aid in the rapid response to emerging viral pathogens.

## Data Availability

All sequencing data referenced have been provided as FASTQ files within the Supplemental Data.

## Supplementary Materials

The following supporting information can be downloaded at https://www.mdpi.com/article/10.3390/v16071064/s1, Table S1: ViBE-Seq RT Adaptors and PCR Primers. Table S2: Comparison Between ViBE-Seq and Current RACE Methods. Data S1: Sequencing Files for Analysis.
